# Physical Activity and the Perceived Neighbourhood Environment — Looking at the Association the Other Way Around

**DOI:** 10.3390/ijerph110808093

**Published:** 2014-08-08

**Authors:** Birgit Wallmann-Sperlich, Ingo Froboese, Peter Schantz

**Affiliations:** 1 Institute of Sport Science, Julius-Maximilians University, Würzburg, D-97082 Würzburg, Germany; 2 Institute of Health Promotion and Clinical Movement Science, German Sport University, D-50933 Cologne, Germany; E-Mail: froboese@dshs-koeln.de; 3 The Research Unit for Movement, Health and Environment, The Swedish School of Sport and Health Sciences, GIH, SE-11486 Stockholm, Sweden; E-Mail: peter.schantz@gih.se; 4 Department of Health Sciences, Mid Sweden University, SE-83125 Östersund, Sweden

**Keywords:** physical activity, perceived physical environment, sex, association, socio-demographic, correlates, Europe, Germany, transport-related physical activity, recreation-related physical activity

## Abstract

The association between physical activity (PA) and variables of the perceived environment mainly originate from cross-sectional studies that introduced the idea that the environment influences the PA level of residents. However, the direction of cause and effect has not been solved with finality. The aim of this study was to investigate whether residents’ perception of their proximate environment differs depending on their level of PA in transport and recreation. We conducted a cross-sectional survey with residents of six different parts of the city of Cologne, Germany. The sample of 470 adults (52.8% females; mean age = 35.5 ± 13.8 years) filled in the Global Physical Activity Questionnaire (GPAQ), as well as the European Environmental Questionnaire ALPHA. To distinguish between residents with “low” and “high” PA, we split the samples into two on the basis of the specific median in transport- and recreation-related PA. In the “high”* vs.* “low” PA group of the overall sample, we noted 4%–16% more “PA favourable” environmental perceptions in seven of the 15 environmental variables. Multiple linear regression analyses were performed to investigate associations of socio-demographic correlates and transport- and recreation-related PA on the dependent variables of the environmental perception. In this case, levels of PA were significant predictors for eight of the 15 items concerning environmental perceptions. Thus, the present study introduces the idea that residents with higher levels of transport and recreational PA may perceive their environment in a more “PA-favourable” way than residents with lower levels.

## 1. Introduction

Promoting physical activity (PA) has evolved into a major public health priority in many countries all over the world [1]. There is growing evidence that the neighbourhood environment can play an important role in hindering or stimulating PA [2,3]. Studies that have been based on subjective measures (perceptions) indicate that a neighbourhood design with nearby destinations, transport environments (access to and quality of sidewalks and bike lanes, connectivity, networks), mixed land use, residential density, recreational resources and aesthetics can affect transport and recreation-related PA [2,4,5]. 

To date, however, most studies are based on cross-sectional findings, which indicate the statistical association between environmental factors and PA, but do not provide us with evidence of a causal relationship [5]. Thus, most research provides a one-directional view of how the environment is associated with PA by investigating environmental correlates of PA [2,3,6]. 

The normal bases for these studies are population-based samples reporting on their own neighbourhoods and on their own PA levels. The underlying assumption has been that the perception of items in the neighbourhoods is independent of levels of the participants’ PA. If, on the other hand, levels of PA affect perceptions of neighbourhoods, then it is important to establish to what extent that is so. 

So far, indications that levels of PA may affect perceptions have been presented in few studies examining changes in environmental perceptions over time [7,8,9]. In one of the first prospective studies, which was designed to examine associations of changes in adults’ environmental perceptions with changes in neighbourhood walking behaviour, the authors reported gender-specific environment-behaviour relationships [7]. Ries and colleagues have documented that during two individual-level PA interventions, the perception of facility and home equipment availability increased [9]. The results of a “3000 steps more per day” PA intervention showed that after the intervention the participants perceived a shorter distance to local facilities, a higher availability of bike lanes and infrastructures, better maintenance of infrastructure, a better network and a safer traffic situation in their neighbourhood environment [8]. In all the mentioned studies, the neighbourhood environment remained unaltered. However, two of the abovementioned studies [7,8] did not include control groups, so it is possible that learning effects, weather effects* etc.* might be involved. 

Although these study results do not allow us to draw a conclusion about the direction of cause and effect and why the environmental perception changed, they strengthen the view that the causal relationship of the perceived neighbourhood environment and PA has not been solved with finality. It has been argued that the perception of the environment can be influenced by the PA level and that it is possible that increased levels of PA might influence the participants’ perceptions of the environment. It might be expected that a higher exposure to the environment through increased PA will lead to a more informed perception of the environment. To our knowledge, no studies to date have investigated how the environmental perception is associated with levels of PA in transport and recreation, which would indeed be of interest and may further our understanding of the interaction of the perception of the neighbourhood environment and PA behaviour. This leads us to the question: do residents with a higher PA level in transport and recreation perceive their environment in a different way from residents living in the same neighbourhood but having a low PA level in transport and recreation? Thus, the aim of the present study is to analyse residents’ perception of the same proximate environment and how it may relate to their levels of PA in transport and recreation. 

## 2. Methods 

### 2.1. Study Design

**Figure 1:**
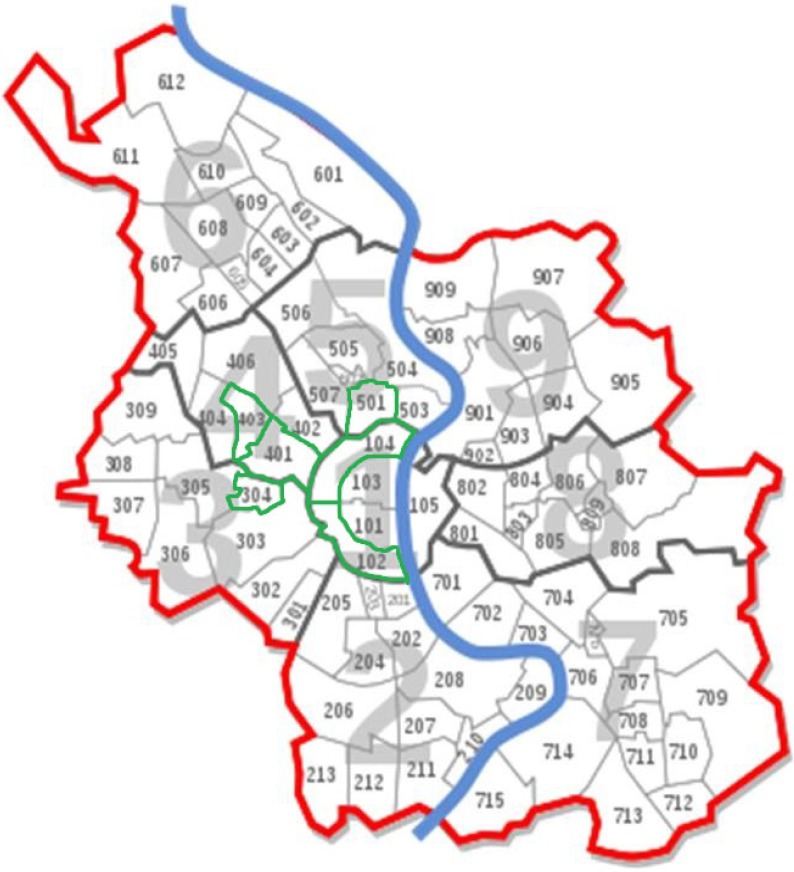
City districts of Cologne [11]. The selected parts of the city (framed in green) were: 403—Bickendorf, 304—Braunsfeld, 401—Ehrenfeld, 104—Neustadt-North, 102—Neustadt-South and 501—Nippes.

We conducted a cross-sectional survey in different parts of the city of Cologne in Germany. Cologne is subdivided into nine city districts with all in all 85 parts (see Figure 1). Participants filled in a self-reported questionnaire on PA and attributes of the perceived environment and gave information on their postal code and part of the city. The recruitment of participants took place between October 2010 and April 2011. In the sample recruitment procedure, research assistants distributed the questionnaire to volunteer participants residing in the selected parts of the city and recruited at such local sites as supermarkets, as well as in neighbourhoods by going from house to house. All study procedures were in accordance with the guidelines of the Ethics Committee of the German Sport University in Cologne, Germany. In line with the aim of the present study, the inclusion criteria for selecting particular parts of the city were (1) a subsample of n ≥ 60 and (2) an area of < 4 km^2^. Six parts of the city satisfied these criteria. Statistical data for Cologne and the selected parts of the city are presented in Table 1.

**Table 1:** Statistical data for Cologne and selected city districts [10].

Characteristics	Cologne	Bickendorf	Braunsfeld	Ehrenfeld	Neustadt-North	Neustadt-South	Nippes
Size of city area in km^2^	405.17	2.31	1.68	3.72	3.49	2.82	3.00
Percentage of recreational area in %	10.4	6.7	1.2	4.3	23.1	16.0	22.6
Inhabitants per km^2^	2557	7208	6383	9684	8215	13,564	11,587
Inhabitants in total	1,036,117	16,632	10,697	36,008	28,665	38,296	34,705
Female, %	51.2	50.9	53.4	49.9	49.4	49.9	51.9
Mean age in city district	41.9	40.1	43.6	39.2	41.1	38.7	40.3
Percentage of people with migration background	33.8	42.6	20.5	34.3	27.4	26.7	29.0
Percentage of single or double houses in the city district	59.6	54.3	39.1	17.4	9.7	7.0	24.6
Number of flats	541,692	7940	6286	20,859	19,049	24,370	19,557
Size of living space per inhabitant	37.6	32.9	45.9	34.1	42.3	38.5	36.8
Number of cars per 1000 inhabitants from the age of 18 years	493	404	580	391	487	353	361
Rate of unemployment	8.6	11.7	5.0	8.9	4.8	5.8	5.8

### 2.2. Study Population

In total, we received 1482 filled-in questionnaires from 79 parts of the city (49.9% females; mean age = 36.4 (SD = 14.4)). From the six selected parts of the city, we received 562 filled-in questionnaires. We excluded respondents because of missing information on sex (n = 4) and age (n = 9). In addition, 60 participants were eliminated from the analyses according to the GPAQ scoring protocol [12] and 19 participants because of missing answers concerning the perception of the environment. In total, due to data cleansing, we lost 16.4% of the participants. We analysed the data for 470 adults (52.8% females, mean age = 35.5; SD = 13.8) living in the six different parts of the city. Sample characteristics including socio-demographic information are shown in Table 2. 

**Table 2:** Characteristics of overall sample and of subsample in selected city districts.

Characteristics		All	Bickendorf	Braunsfeld	Ehrenfeld	Neustadt-North	Neustadt-South	Nippes
*All* (n)		470	65	83	90	61	100	71
	*Male* (n; %)	222 (47.2)	30 (46.2)	41 (49.4)	45 (50.0)	30 (49.2)	41 (41.0)	35 (49.3)
	*Female* (n; %)	248 (52.8)	35 (53.8)	42 (50.6)	45 (50.0)	31 (50.8)	59 (59.0)	36 (50.7)
*Age* (years)		35.5 ± 13.8	40.0 ± 15.7	30.6 ± 10.4	34.0 ± 11.9	33.3 ± 12.7	36.2 ± 11.7	40.0 ± 18.2
*BMI* (kg × m^−2^)		23.7 ± 3.2^ a^	23.1 ± 3.2	23.6 ± 2.9 ^b^	24.9 ± 4.1 ^c^	23.5 ± 2.9 ^d^	23.6 ± 3.1 ^e^	23.8 ± 2.8
*Educational level (n; %)*								
	No graduation	9 (1.9)	1 (1.5)	1 (1.2)	3 (3.3)	1 (1.6)	3 (3.0)	-
	10 years	53 (11.3)	20 (30.8)	4 (4.8)	4 (4.4)	7 (11.5)	15 (15.0)	3 (4.2)
	12 years	98 (20.9)	18 (27.7)	11 (13.3)	24 (26.7)	12 (19.7)	21 (21.0)	12 (16.9)
	13 years	158 (33.6)	16 (24.6)	42 (50.6)	28 (31.1)	19 (31.1)	26 (26.0)	27 (38.0)
	≥University degree	140 (29.8)	8 (12.3)	25 (30.1)	22 (24.4)	21 (34.4)	35 (35.0)	29 (40.8)
	Missing values	12 (2.6)	2 (3.1)	-	9 (10.0)	1 (1.6)	-	-
*Income groups household net income/month (n; %)*								
	<€1000	134 (28.5)	20 (30.8)	36 (43.4)	18 (20.0)	24 (39.3)	23 (23.0)	13 (18.3)
	€1000–€2000	196 (41.7)	38 (58.5)	31 (37.3)	38 (42.2)	20 (32.8)	25 (25.0)	44 (62.0)
	>€2000	85 (18.1)	1 (1.5)	14 (16.9)	17 (18.9)	10 (16.4)	29 (29.0)	14 (19.7)
	Missing values	55 (11.7)	6 (9.2)	2 (2.4)	17 (18.9)	7 (11.5)	23 (23.0)	-

Notes: ^**a**^ Sample size for BMI response → n = 430; **^b^** sample size for BMI response → n = 82; **^c^** sample size for BMI response → n = 71; **^d^** sample size for BMI response → n = 54; **^e^** sample size for BMI response → n = 87.

### 2.3. Measurements

#### 2.3.1. Physical Activity

We used the Global Physical Activity Questionnaire (GPAQ) [13] to assess the prevalence of PA in a usual week. The 16 questions of the GPAQ were designed to measure PA in three domains: work (paid and unpaid), transport (*i.e.*, walking and cycling to get to and from places) and recreational activities [13]. In the work and recreation domains, information on the frequency and duration of vigorous intensity PA, as well as moderate intensity PA, was obtained. For the transport domain, information on walking and cycling activities was included without differentiation between the intensities. All reported PA was supposed to last for at least 10 continuous minutes. 

Weekly minutes of moderate and vigorous intensity activity were calculated separately by multiplying the number of days per week by the duration for an average day. Reported minutes per week in each category were multiplied by the metabolic equivalent (MET), which is generally used for expressing the intensity of PA independently of body weight. Four METs corresponded to the time spent in moderate intensity activities and eight METs to the time spent in vigorous intensity activities [12]. As outcome measures, MET min/week at moderate and vigorous intensity in work and recreation and moderate-intensity transport-related PA were obtained. For further analyses, we only summed up the MET min/week for transport- and recreation-related PA without adding the work-related PA. All GPAQ data were checked for possible data entry errors by using the provided WHO CleanRecode program [14]. 

Validity and reliability have been assessed previously in nine different countries. Concurrent validity between the International Physical Activity Questionnaire (IPAQ), a previously validated and accepted measure of physical activity, and the GPAQ showed a moderate to strong positive relationship (range 0.45 to 0.65) and reliability was of moderate to substantial strength (kappa, 0.67 to 0.73; Spearman’s rho, 0.67 to 0.81) [15]. 

#### 2.3.2. Environmental Variables

The assessment of the perceived environment was self-administered using the German version of the European Environmental Questionnaire, ALPHA. This questionnaire consisted of nine themes concerning the neighbourhood (types of residences, distances to local facilities (both five-point scales), walking or cycle infrastructure, maintenance of infrastructure, neighbourhood safety, pleasure and aesthetics of the neighbourhood, cycling and walking network (all four-point scales), home environment, workplace or study environment (both dichotomised “yes”, “no”)) with a total of 49 items that were summed into 15 theme scores and have been described elsewhere [16,17]. The item “distance” displays with a higher score a longer distance to facilities, indicating a less supportive environment for PA. Subsequently, we recoded the item ‘distance’ so that a higher score referred to a more supportive environment for PA. The reliability of the instrument has been demonstrated (ICC 0.71–0.87) and it was translated from English into German, followed by cognitive testing [17]. The context validity and reliability of the German translation has been confirmed [18].

#### 2.3.3. Socio-Demographic Variables

The demographic variables measured self-reported age, sex and the body mass index (BMI), calculated using self-reported body weight and body height according to the formula BMI = body weight (kg) × (body height (m))^-2^. For further socio-demographic variables, education and income levels were included. The educational level was categorised into the following levels based on the German school system: no graduation, 10 years of education, 12 years of education, 13 years of education, university degree or higher. Monthly household net income was assessed in nine categories and summarised in three groups: <€1000, €1000–€2000 and >€2000. 

### 2.4. Data Analyses

All analyses were performed using IBM SPSS Statistics 22 and Microsoft Excel 2010 for Windows. Medians were calculated for transport- and recreation-related PA in the overall sample as well as in the six selected parts of the city. To distinguish between residents with low and high transport- and recreation-related PA in the different parts of the city, we split each subsample into two on the basis of the specific median in transport- and recreation-related PA. To avoid “in-between” city district variation [19] in the further analyses for the overall sample, we calculated the relative environmental perceptions (individual perception involving a specific environmental theme/median perception involving a specific environmental theme within the part of the city) and the relative PA values (individual PA/median PA within the part of the city). Differences in the environmental perception between residents with low and high PA, as well as for the 1st and 4th quartile of PA, were calculated using an independent *t*-test in the overall sample (relative values), as well as for each part of the city (absolute values). To describe the relationship between residents with low and high PA, we also calculated the ratio of the environmental perception following the formula: high PA/low PA or 4th quartile/1st quartile. 

Multiple linear regression analyses were performed to investigate associations of socio-demographic correlates and transport- and recreation-related PA with the dependent variables of the environment perception in the 15 themes for the overall sample and each city district separately. We chose the forced entry method and included the following socio-demographic variables: age (continuous variable), BMI (continuous variable), education (four categories) and income level (three categories) and included transport- and recreation-related PA as a continuous variable. To overcome the influence of different environments in the overall sample, we used, as the dependent variable, the relative environmental perception and, as independent variables, the relative PA values. Statistical significance was set at a level of 0.05.

## 3. Results 

### 3.1. Descriptive Measures of PA

The overall median in transport and recreation-related PA was 330 MET minutes per week with a range between 233 and 510 MET minutes per week for the selected city districts (see Table 3). For the dichotomised groups low and high PA in transport and recreation, the average values were 165 *vs*. 510 MET min per week, with an overall median ratio of high/low PA of 3.09. The corresponding ratios between the city districts range between 2.4 for Nippes and 7.02 for Ehrenfeld, showing a great difference between the median of the low PA and the high PA groups.

### 3.2. Descriptive Measures and Differences in Environmental Perception

The relative means and standard deviations, as well as the ratio between the relative environmental perception of residents with low PA and high PA in the overall sample, are presented in Table 4. The ratios between the dichotomised samples vary between 0.97 and 1.16. The meaning of this is that with ratios higher than 1.0, a more “PA-friendly” environmental perception is noted in residents with high levels of PA compared to residents with low levels of PA. In seven out of 15 environmental scores, residents with high PA perceive what is considered to be a more PA-friendly neighbourhood. The following differences were noted in “distance” (1.05), “availability of bike lanes” (1.07), “availability of infrastructure” (1.04), “network” (1.06), “connectivity” (1.06), “home environment” (1.13) and “work or study environment” (1.16). When we look at the comparison between the 1st and 4th quartiles of relative transport and recreation PA, residents in the 4th quartile indicate essentially the same differences, but with increased magnitudes. 

**Table 3:** PA in transport and recreation (MET min × week^-1^) of the overall sample and of the subsample in selected city districts.

PA Characteristics		All (n = 470)	Bickendorf (n = 65)	Braunsfeld(n = 83)	Ehrenfeld (n = 90)	Neustadt-North (n = 61)	Neustadt-South (n = 100)	Nippes (n = 71)
PA in transport and recreation (Mdn) (25^th^; 75^th^)		330 (160; 510)	300 (130; 450)	510 (300; 870)	233 (50; 365)	240 (120; 405)	300 (180; 446)	360 (255; 630)
	Male	308 (159; 513)	285 (120; 450)	480 (240; 1200)	240 (45; 360)	230 (80; 488)	300 (165; 450)	360 (270; 630)
	Female	335 (163; 508)	300 (180; 500)	515 (401; 698)	225 (55; 399)	240 (120; 360)	300 (180; 435)	390 (244; 615)
Low PA in transport and recreation * (Mdn) (25^th^; 75^th^)		165 (80; 240)	165 (100; 240)	330 (180; 465)	50 (25; 123)	120 (12; 180)	180 (110; 250)	263 (150; 320)
High PA in transport and recreation ** (Mdn) (25^th^; 75^th^)		510 (420; 740)	460 (383; 570)	870 (603; 1425)	360 (328; 550)	450 (360; 537)	450 (390; 720)	630 (480; 840)

Notes: Mdn—Median; * 1st and 2nd quartiles of the sample regarding, respectively, the overall and city district-specific median split in PA in transport and recreation; ** 3rd and 4th quartiles of the sample regarding, respectively, the overall and city district-specific median split in PA in transport and recreation.

**Table 4:** Results of independent t-test for the relative transport and recreation PA differences (overall median split; 1st quartile vs 4th quartile) in the relative perception of the themes of the neighbourhood environment (mean ± standard deviation).

PA Median Split Groups	x ± s	PA Quartile Groups	x ± s
Density score			
All	1.0 ± 0.27		
Low PA	0.99 ± 0.25	1st quartile	1.01 ± 0.26
High PA	1.01 ± 0.29	4th quartile	1.01 ± 0.31
Ratio ^†^	1.02	Ratio ^††^	1.00
Distance score			
All	1.0 ± 0.10		
Low PA	0.98 ± 0.08	1st quartile	0.96 ± 0.08
High PA	1.03 ± 0.10	4th quartile	1.04 ± 0.10
Ratio ^†^	1.05 ***	Ratio ^††^	1.08 ***
Availability of sidewalks			
All	1.0 ± 0.17		
Low PA	0.99 ± 0.16	1st quartile	0.98 ± 0.16
High PA	1.01 ± 0.18	4th quartile	1.03 ± 0.19
Ratio ^†^	1.02	Ratio ^††^	1.05
Availability of bike lanes			
All	1.0 ± 0.23		
Low PA	0.96 ± 0.22	1st quartile	0.96 ± 0.22
High PA	1.03 ± 0.25	4th quartile	1.07 ± 0.25
Ratio ^†^	1.07 **	Ratio ^††^	1.11 **
Availability of infrastructure			
All	1.0 ± 0.15		
Low PA	0.98 ± 0.14	1st quartile	0.97 ± 0.13
High PA	1.02 ± 0.17	4th quartile	1.05 ± 0.18
Ratio ^†^	1.04 **	Ratio ^††^	1.08 ***
Maintenance of infrastructure			
All	1.0 ± 0.18		
Low PA	0.99 ± 0.17	1st quartile	0.97 ± 0.18
High PA	1.01 ± 0.19	4th quartile	1.01 ± 0.21
Ratio ^†^	1.02	Ratio ^††^	1.04
Total safety			
All	1.0 ± 0.15		
Low PA	1.0 ± 0.15	1st quartile	0.99 ± 0.15
High PA	0.99 ± 0.15	4th quartile	0.99 ± 0.16
Ratio ^†^	0.99	Ratio ^††^	1.00
Safety crime			
All	1.0 ± 0.17		
Low PA	1.0 ± 0.18	1st quartile	0.99 ± 0.19
High PA	1.0 ± 0.17	4th quartile	1.0 ± 0.18
Ratio ^†^	1.00	Ratio ^††^	1.01
Safety traffic			
All	1.0 ± 0.19		
Low PA	1.0 ± 0.18	1st quartile	1.0 ± 0.19
High PA	0.99 ± 0.19	4th quartile	0.98 ± 0.20
Ratio ^†^	0.99	Ratio ^††^	0.98
Pleasure			
All	1.0 ± 0.16		
Low PA	1.01 ± 0.16	1st quartile	1.0 ± 0.17
High PA	0.99 ± 0.17	4th quartile	0.98 ± 0.18
Ratio ^†^	0.99	Ratio ^††^	0.98
Aesthetics			
All	1.0 ± 0.17		
Low PA	1.02 ± 0.16	1st quartile	1.0 ± 0.17
High PA	0.99 ± 0.17	4th quartile	0.98 ± 0.18
Ratio ^†^	0.97	Ratio ^††^	0.98
Network			
All	1.0 ± 0.15		
Low PA	0.97 ± 0.15	1st quartile	0.95 ± 0.15
High PA	1.03 ± 0.15	4th quartile	1.04 ± 0.15
Ratio ^†^	1.06 ***	Ratio ^††^	1.09 ***
Connectivity			
All	1.0 ± 0.16		
Low PA	0.97 ± 0.16	1st quartile	0.95 ± 0.17
High PA	1.03 ± 0.16	4th quartile	1.04 ± 0.17
Ratio ^†^	1.06 ***	Ratio ^††^	1.09 ***
Home			
All	1.0 ± 0.41		
Low PA	0.94 ± 0.40	1st quartile	0.89 ± 0.37
High PA	1.06 ± 0.42	4th quartile	1.15 ± 0.43
Ratio ^†^	1.13 **	Ratio ^††^	1.29 ***
Work/Study			
All	0.97 ± 0.42		
Low PA	0.90 ± 0.38	1st quartile	0.89 ± 0.40
High PA	1.04 ± 0.45	4th quartile	1.07 ± 0.49
Ratio ^†^	1.16 **	Ratio ^††^	1.20 **

Notes: sample size for all neighbourhood themes except “work/study”: All (n = 470); low PA (n = 239); high PA (n = 231); 1st quartile (n = 122); 4th quartile (n = 116); sample size for the neighbourhood theme “work/study”: All (n = 387); low PA (n = 190); high PA (n = 197); 1st quartile (n = 99); 4th quartile (n = 103); **^†^** Ratio = (high PA/low PA ); **^††^** Ratio = (4th/1st); ** *p* < 0.01; *** *p* < 0.001.

In the six different districts of the city there was the following distribution of the environmental perception ratio (significantly more “PA-friendly” districts/non-significant districts/significantly less “PA-friendly” districts): density score (2/3/1), distance score (2/4/0), availability of sidewalks (1/5/0), availability of bike lanes (3/3/0), availability of infrastructure (1/5/0), maintenance of infrastructure (1/5/0), total safety (1/4/1), safety, crime (1/4/1), safety, traffic (1/5/0), pleasure (0/5/1), aesthetics (0/5/1), network (3/3/0), connectivity (2/4/0), home environment (1/5/0) and work or study environment (3/3/0) (see Table 5). 

**Table 5:** Results of independent t-test for transport and recreation PA differences (city district-specific median split) in the perception of the themes of the neighbourhood environment (mean ± standard deviation).

PA median split groups	Bickendorf (n = 65)	Braunsfeld (n = 83)	Ehrenfeld (n = 90)	Neustadt-North (n = 61)	Neustadt-South (n = 100)	Nippes (n = 71)
Density score						
All	193.0 ± 38.1	188.4 ± 51.0	177.0 ± 47.4	126.0 ± 33.9	164.8 ± 47.0	173.1 ± 51.7
Low PA	192.2 ± 39.6	201.6 ± 45.2	162.4 ± 39.3	115.3 ± 14.7	173.0 ± 48.4	172.0 ± 53.3
High PA	193.9 ± 36.8	175.0 ± 53.6	191.6 ± 50.5	138.6 ± 44.6	155.5 ± 44.1	174.2 ± 50.8
Ratio ^†^	1.01	0.87 *	1.18 **	1.20 **	0.90	1.01
Distance score						
All	24.4 ± 2.3	34.0 ± 3.4	31.2 ± 4.2	33.4 ± 2.8	36.8 ± 2.0	36.1 ± 3.3
Low PA	24.0 ± 2.3	33.3 ± 3.6	29.0 ± 2.4	32.4 ± 2.4	36.8 ± 1.6	35.7 ± 3.2
High PA	25.0 ± 2.2	34.4 ± 3.2	33.4 ± 4.4	34.6 ± 2.9	36.7 ± 2.4	36.5 ± 3.3
Ratio ^†^	1.04	1.03	1.15 ***	1.07 **	1.00	1.02
Availability of sidewalks						
All	5.9 ± 1.0	6.0 ± 1.0	6.1 ± 1.2	5.7 ± 0.9	6.6 ± 0.9	6.0 ± 1.2
Low PA	5.9 ± 1.2	5.7 ± 0.7	6.2 ± 1.2	5.7 ± 0.9	6.6 ± 0.8	5.9 ± 1.0
High PA	5.9 ± 0.8	6.3 ± 1.1	6.0 ± 1.1	5.8 ± 1.0	6.6 ± 1.0	6.1 ± 1.3
Ratio ^†^	1.00	1.11 *	0.97	1.02	1.00	1.03
Availability of bike lanes						
All	4.4 ± 1.3	6.0 ± 1.3	5.8 ± 1.3	4.7 ± 1.4	5.9 ± 1.0	5.6 ± 1.3
Low PA	4.3 ± 1.3	5.6 ± 1.2	5.5 ± 1.2	4.3 ± 1.1	6.0 ± 0.7	5.5 ± 1.4
High PA	4.6 ± 1.3	6.3 ± 1.4	6.0 ± 1.3	5.1 ± 1.5	5.8 ± 1.3	5.7 ± 1.1
Ratio^ †^	1.07	1.13 *	1.09 *	1.19 *	0.97	1.03
Availability of infrastructure						
All	10.3 ± 1.5	12.0 ± 1.9	11.9 ± 1.9	10.4 ± 1.8	12.5 ± 1.5	11.6 ± 2.0
Low PA	10.1 ± 1.3	11.4 ± 1.5	11.8 ± 1.7	10.0 ± 1.4	12.5 ± 1.2	11.4 ± 2.1
High PA	10.5 ± 1.6	12.6 ± 1.9	12.0 ± 2.0	10.9 ± 2.2	12.3 ± 1.7	11.8 ± 1.9
Ratio ^†^	1.04	1.11 **	1.02	1.09	0.98	1.04
Maintenance of infrastructure						
All	9.0 ± 1.4	8.3 ± 1.6	7.3 ± 1.7	8.2 ± 1.6	8.9 ± 1.3	8.7 ± 1.4
Low PA	9.1 ± 1.3	8.6 ± 1.5	6.7 ± 1.4	8.0 ± 1.6	9.1 ± 0.9	8.4 ± 1.5
High PA	8.7 ± 1.5	8.1 ± 1.8	7.9 ± 1.7	8.4 ± 1.6	8.7 ± 1.5	9.0 ± 1.3
Ratio ^†^	0.96	0.94	1.18 ***	1.05	0.96	1.07
Total safety						
All	15.9 ± 2.0	20.1 ± 3.3	17.7 ± 2.5	17.3 ± 2.4	18.2 ± 2.4	17.3 ± 3.2
Low PA	15.8 ± 2.3	20.4 ± 3.4	17.1 ± 2.2	16.8 ± 1.7	18.8 ± 2.5	17.7 ± 3.3
High PA	15.9 ± 1.7	19.7 ± 3.2	18.3 ± 2.7	17.8 ± 2.9	17.5 ± 2.3	16.8 ± 3.1
Ratio ^†^	1.01	0.97	1.07 *	1.06	0.93 *	0.95
Safety, crime						
All	8.7 ± 1.4	10.2 ± 1.5	8.8 ± 1.5	8.2 ± 1.3	9.2 ± 1.6	8.5 ± 2.0
Low PA	8.6 ± 1.5	10.4 ± 1.5	8.6 ± 1.5	7.8 ± 1.0	9.6 ± 1.5	8.8 ± 2.2
High PA	8.9 ± 1.2	10.0 ± 1.5	9.0 ± 1.5	8.7 ± 1.5	8.7 ± 1.6	8.3 ± 1.7
Ratio ^†^	1.03	0.96	1.05	1.12 **	0.91 *	0.94
Safety, traffic						
All	7.2 ± 1.6	9.9 ± 2.0	8.9 ± 1.7	9.0 ± 1.6	9.0 ± 1.3	8.7 ± 1.8
Low PA	7.3 ± 1.6	10.0 ± 2.1	8.5 ± 1.5	9.0 ± 1.4	9.1 ± 1.4	9.0 ± 1.7
High PA	7.0 ± 1.7	9.7 ± 2.0	9.3 ± 1.7	9.1 ± 1.8	8.8 ± 1.0	8.5 ± 2.0
Ratio ^†^	1.04	0.97	1.09 *	1.01	0.97	0.94
Pleasure						
All	9.5 ± 2.0	12.7 ± 2.0	10.4 ± 1.9	12.1 ± 1.4	12.1 ± 1.4	11.2 ± 2.0
Low PA	9.6 ± 1.9	12.8 ± 1.8	10.3 ± 2.1	12.3 ± 1.3	12.6 ± 1.2	11.2 ± 2.0
High PA	9.5 ± 2.2	12.6 ± 2.2	10.5 ± 1.7	11.9 ± 1.6	11.6 ± 1.5	11.1 ± 2.1
Ratio ^†^	0.99	0.98	1.02	0.97	0.92 ***	0.99
Aesthetics						
All	7.1 ± 1.4	9.5 ± 1.5	8.2 ± 1.6	9.2 ± 1.1	9.1 ± 1.2	8.6 ± 1.5
Low PA	7.1 ± 1.3	9.5 ± 1.4	8.3 ± 1.7	9.3 ± 1.1	9.6 ± 0.9	8.6 ± 1.5
High PA	7.1 ± 1.5	9.6 ± 1.6	8.2 ± 1.5	9.1 ± 1.1	8.6 ± 1.2	8.6 ± 1.6
Ratio ^†^	1.00	1.01	0.99	0.98	0.90 ***	1.00
Network						
All	12.3 ± 1.9	11.8 ± 1.9	11.5 ± 1.9	12.2 ± 1.6	11.6 ± 1.7	11.9 ± 1.9
Low PA	12.3 ± 2.0	11.1 ± 1.6	11.1 ± 1.7	12.1 ± 1.6	11.2 ± 1.7	11.6 ± 2.0
High PA	12.3 ± 1.8	12.5 ± 1.9	11.9 ± 2.0	12.4 ± 1.6	12.0 ± 1.6	12.2 ± 1.8
Ratio ^†^	1.00	1.13 **	1.07 *	1.02	1.07 *	1.05
Connectivity						
All	9.0 ± 1.5	8.8 ± 1.4	8.4 ± 1.6	8.8 ± 1.3	8.8 ± 1.2	8.7 ± 1.5
Low PA	8.9 ± 1.6	8.5 ± 1.2	8.0 ± 1.6	8.6 ± 1.4	8.7 ± 1.1	8.5 ± 1.6
High PA	9.3 ± 1.4	9.1 ± 1.6	8.8 ± 1.6	9.1 ± 1.2	9.0 ± 1.2	9.0 ± 1.4
Ratio ^†^	1.04	1.12 *	1.10 *	1.06	1.03	1.06
Home						
All	2.8 ± 0.8	3.1 ± 1.0	2.9 ± 1.1	2.2 ± 1.1	2.2 ± 0.9	2.4 ± 1.3
Low PA	2.6 ± 0.7	2.9 ± 1.0	3.0 ± 1.2	1.9 ± 0.9	2.0 ± 1.0	2.3 ± 1.2
High PA	3.0 ± 0.9	3.3 ± 0.9	2.8 ± 1.0	2.6 ± 1.2	2.3 ± 0.9	2.5 ± 1.3
Ratio ^†^	1.15	1.14	0.93	1.37 **	1.15	1.09
Work/Study						
All	3.2 ± 1.3	5.4 ± 2.3	3.6 ± 1.5	3.0 ± 1.5	3.5 ± 1.7	4.2 ± 1.5
Low PA	3.4 ± 1.5	4.7 ± 2.1	3.2 ± 1.2	2.8 ± 1.4	3.5 ± 1.6	3.5 ± 0.8
High PA	3.0 ± 1.0	6.1 ± 2.3	4.0 ± 1.7	3.3 ± 1.5	3.5 ± 1.9	4.7 ± 1.7
Ratio ^†^	0.88	1.30 **	1.25 *	1.18	1.00	1.34 **

Notes: ^**†**^ Ratio = (high PA/low PA); * *p* < 0.05; ** *p* < 0.01; *** *p* < 0.001.

### 3.3. Multiple Regression Analyses for the Dependent Variables of Environmental Perception 

Table 6 presents the significant correlates associated with the environmental perception, as well as the R^2^ values of multiple regression analyses for all subjects, as well as separately for the different city districts. In the overall model, higher levels of transport and recreation-related PA predicted higher levels of perceptions of the following environmental variables: “distance”, “availability of sidewalks”, “availability of bicycle lanes”, “availability of infrastructure”, “network”, “connectivity”, “home environment” and “work/study environment”. For the themes of “density”, “availability of bike lanes”, “maintenance of infrastructure”, “total safety”, “safety, crime”, “safety, traffic” and “pleasure”, no distinct direction of perception concerning the PA-levels was documented. For the theme “aesthetics”, a negative association was noted, meaning that with higher PA levels, the environment is perceived as being less aesthetic.

**Table 6:** Significant standardised β-values and all R^2^-values of multiple regression analyses of the dependent variables of environmental perception and the independent variables, transport- and recreation-related PA, as well as socio-demographic variables (sex, age, education and income) in the overall sample and in the selected city districts of Bickendorf, Braunsfeld, Ehrenfeld, Neustadt-Nord, Neustadt-Süd and Nippes.

Themes of Neighbourhood	Relative Values for All (n_1_ = 407; n_2_ = 338)	Bickendorf (n_1 _= 59; n_2 _= 50)	Braunsfeld (n_1 _= 81; n_2 _= 70)	Ehrenfeld (n_1 _= 66; n_2 _= 62)	Neustadt-North (n_1 _= 53; n_2 _= 45)	Neustadt-South (n_1 _= 77; n_2 _= 57)	Nippes (n_1 _= 71; n_2 _= 54)
Density score	R^2 ^= 0.01	R^2 ^= 0.04	Sex (−0.20) * PA (−0.40) *** R^2 ^= 0.25	Sex (−0.35) ** PA (0.33) ** R^2 ^= 0.23	R^2 ^= 0.10	Age (−0.27) * PA (−0.46) *** R^2 ^= 0.31	R^2 ^= 0.04
Distance score	Inc (−0.11) * PA (0.16) ** R^2 ^= 0.06	R^2 ^= 0.04	R^2 ^= 0.11	PA (0.25) * R^2 ^= 0.17	R^2 ^= 0.14	Edu (0.33) *, Inc (−0.31) * R^2 ^= 0.11	R^2 ^= 0.07
Availability of sidewalks	PA (0.15) ** R^2 ^= 0.02	R^2 ^= 0.15	PA (0.30) * R^2 ^= 0.11	Sex (0.31) * R^2 ^= 0.14	R^2 ^= 0.05	Edu (−0.34) * R^2 ^= 0.08	PA (0.25) * R^2 ^= 0.17
Maintenance of infrastructure	R^2 ^= 0.01	R^2 ^= 0.04	R^2 ^= 0.04	PA (0.44) ** R^2 ^= 0.20	R^2 ^= 0.10	PA (−0.38) ** R^2 ^= 0.23	R^2 ^= 0.05
Availability of bike lanes	PA (0.15) ** R^2 ^= 0.04	Sex (−0.31) * R^2 ^= 0.14	PA (0.29) * R^2 ^= 0.15	PA (0.30) * R^2 ^= 0.12	R^2 ^= 0.11	PA (−0.29) * R^2 ^= 0.13	PA (0.31) * R^2 ^= 0.12
Availability of infrastructure	Age (0.10) * PA (0.19) *** R^2 ^= 0.05	Sex (−0.35) * R^2 ^= 0.16	PA (0.37) ** R^2 ^= 0.20	R^2 ^= 0.14	R^2 ^= 0.10	R^2 ^= 0.07	PA (0.34) * R^2 ^= 0.18
Total safety	Age (−0.14) ** R^2 ^= 0.03	Inc (−043) ** R^2 ^= 0.19	PA (−0.27) * R^2 ^= 0.12	PA (0.37) ** R^2 ^= 0.16	PA (0.33) * R^2 ^= 0.15	Age (−0.42) ** R^2 ^= 0.22	Age (−0.13) R^2 ^= 0.10
Safety, crime	Age (−0.13) * R^2 ^= 0.04	R^2 ^= 0.12	R^2 ^= 0.13	PA (0.30) * R^2 ^= 0.13	PA (0.35) * R^2 ^= 0.13	Age (−0.32) * R^2 ^= 0.16	R^2 ^= 0.11
Safety, traffic	R^2 ^= 0.01	Inc (−0.33) * R^2 ^= 0.13	PA (−0.27) * R^2 ^= 0.11	PA (0.29) * R^2 ^= 0.08	Inc (0.34) * R^2 ^= 0.15	Age (−0.37) ** R^2 ^= 0.14	R^2 ^= 0.10
Pleasure	R^2 ^= 0.01	R^2 ^= 0.09	R^2 ^= 0.07	R^2 ^= 0.02	R^2 ^= 0.08	PA (−0.30) * R^2 ^= 0.10	R^2 ^= 0.07
Aesthetics	PA (−0.12) * R^2 ^= 0.02	R^2 ^= 0.08	R^2 ^= 0.08	R^2 ^= 0.00	Sex (−0.30) * R^2 ^= 0.11	PA (−0.35) ** R^2 ^= 0.13	R^2 ^= 0.09
Network	PA (0.14) ** R^2 ^= 0.03	R^2 ^= 0.06	PA (0.26) * R^2 ^= 0.11	Inc (−0.26) * R^2 ^= 0.09	R^2 ^= 0.12	Age (0.29) * R^2 ^= 0.15	PA (0.24) * R^2 ^= 0.16
Connectivity	PA (0.13) * R^2 ^= 0.03	R^2 ^= 0.07	R^2 ^= 0.09	Inc (−0.28) * R^2 ^= 0.10	PA (0.33) * R^2 ^= 0.13	R^2 ^= 0.10	R^2 ^= 0.16
Home	Edu (0.15) ** Inc (−0.12) * PA (0.16) ** R^2 ^= 0.06	Sex (−0.32) * PA (0.29) * R^2 ^= 0.21	R^2 ^= 0.04	Sex (0.31) * R^2 ^= 0.11	Edu (0.32) * R^2 ^= 0.23	PA (0.26) * R^2 ^= 0.08	PA (0.32) ** R^2 ^= 0.17
Work/Study	Inc (−0.32) *** PA (0.20) *** R^2 ^= 0.16	R^2 ^= 0.06	Sex (−0.26) * Inc (−0.53) *** PA (0.27) ** R^2 ^= 0.46	Inc (−0.37) ** R^2 ^= 0.19	Sex (0.31) * PA (0.49) ** R^2 ^= 0.23	Inc (−0.42) ** R^2 ^= 0.18	PA (0.35) * R^2 ^= 0.23

Notes: n_1_ = sample size for the neighbourhood perception except the Work/Study perception; n_2_ = sample size for the Work/Study perception; Edu = education; Inc = income; * *p* < 0.05; ** *p* < 0.01; *** *p* < 0.001.

## 4. Discussion 

Previous studies investigating possible environmental correlates of PA have shown that particularly themes that contribute to the concept of walkability, including the three key elements of residential density, land use mix and street connectivity, as well as access to destinations, an activity-friendly transport environment and aesthetics, are all positively related to PA in the different domains [2,5,6]. As a consequence, city and transport planners, as well as political decision-makers, are urged to take these matters into consideration to promote PA [20]. To the best of our knowledge, the present study is one of the first cross-sectional studies to investigate the relationship between PA and the environment from the opposite view,* i.e.*, examining the association between transport- and recreation-related PA and the perception of the environment by residents in six different neighbourhoods. 

Most of the results obtained from the differences between the dichotomised PA groups are supported by the results of the multiple regression analyses controlling for socio-demographic correlates. These indicate that residents with high PA perceive their neighbourhood in what is often considered to be a more “PA-favourable” way with regard to the themes of “distance”, “availability of sidewalks”, “availability of bicycle lanes”, “availability of infrastructure”, “network”, “connectivity”, “home environment” and “work/study environment” compared to the residents with low PA in the similar neighbourhood. The majority of these themes are in accord with the above-mentioned usually stated PA-related environmental correlates [5]. 

These results indicate that a higher PA in transport and recreation may lead to an altered perception of the neighbourhood. This is in line with the results of recent PA intervention studies investigating the effect of increasing PA on the perception of the environment without actually altering it [8,9]. Reasons for the disparities in environmental perceptions might stem from the fact that people with low PA simply have not at all, or only partly, discovered, for example, that local facilities are within walking distance or the existence of the walking and cycling infrastructure, all of which can lead to misperceptions of their neighbourhood environment [21]. Thus, residents with high transport and recreational PA may have higher frequencies of contact with the features of their neighbourhood, so that they are more aware of the existing PA-friendly resources, which may lead to a higher PA-friendly perception of their neighbourhood. 

The present comparisons of perceptions of the environment by participants with the lowest 25% levels of PA with the corresponding highest 25% levels of PA yield the same principal results as those for the dichotomised samples, whereas the magnitude of the differences appears to increase. The sizes of these differences (4%–16% between the dichotomised samples and 8%–29% between the lowest and highest quartile groups of values) can be useful for analytical purposes in future as well as previous studies on these matters. In our opinion, these magnitudes of perceived environmental differences must be exceeded if we are to be able to interpret them as reflecting true differences in environmental features between neighbourhood areas that represent inhabitants with low and high levels of PA. 

Our results also show, however, that these associations of PA were not evident in all neighbourhoods and not for all environmental themes. This may be due to the relatively small number of individuals surveyed in each city district. Another reason for this could be found in the ratios of high PA/low PA for the different city districts. Ehrenfeld, with the highest ratio of 7.02, which is mainly due to the very low median of the low PA group, also exhibits very large differences in the perceptions of the environment. This may indicate that the associations with PA occur with quite low PA levels or stand out more clearly with large differences in the levels of PA. 

The reasons for the inconsistency of the PA association in the theme “density” in different city districts may be a result of a high heterogeneity of buildings in Cologne, where even in small parts of the city, blocks of flats and detached houses are close together. The city district Braunsfeld with 6383 inhabitants per km^2^, which is the least dense district compared to the other city districts (see Table 1) also has the highest level of PA in transport and recreation. Conversely, this finding indicates that a critical mass of residents is required to make an adequate density of shops or an attractive public transport system viable, but the relationship between density and PA may not be linear. Nevertheless, our findings support the conclusion of van Holle* et al.* concerning the inconsistencies in the theme of “density”, namely, that this environmental theme seems to be associated differently in the European context compared to the results from the United States and Australia [6]. To clarify these issues, further European research is necessary.

Regarding the opposite association of “availability of bike lanes” and “maintenance of infrastructure” in Neustadt-South compared to the positive association in the other city districts, an explanation may be found in the specific situation of Neustadt-South that was characterised by extensive construction work in the past few years due to the work on a new underground railway in this area which resulted in obstruction of or damage to many bike lanes. The same explanation can be applied to the single negative association within the environmental themes “aesthetics” and “pleasure” in this district, which is in contrast to other data [22,23]. In this case, residents with a higher transport and recreation-related PA may be more disturbed due to the mentioned cause. 

With respect to “perceived safety”, there are overall mixed findings in the literature concerning an association with PA [2,22,24], which we can also confirm by looking at the results for the different city districts. These inconsistencies might be due to a multifactorial influence on the individual perception of safety where other socio-demographic correlates such as age or household income play a more decisive role. Overall, the socio-demographic correlates do not play a consistent role in environmental perception, which indicates the need for further studies investigating socio-demographic correlates. All in all, future studies are warranted to define the actual causal relationship between PA and the neighbourhood environment, which emphasises the need for longitudinal studies on this matter [5]. 

### Limitations and Strengths

The use of subsamples in six different small neighbourhoods where objective neighbourhood factors are similar was one of the strengths of the study with the aim of investigating differences in the perception of the environment by PA level. However, the sample sizes were small and objective measurement of the neighbourhood environment, e.g., GIS, would have strengthened the analyses and results. Furthermore, a location-specific PA questionnaire that distinguishes between PA within and outside the local neighbourhood and takes account of context-specific PA behaviour, such as recreation- and transport-related walking, e.g. the Neighbourhood Physical Activity Questionnaire [25], would have been useful for interpreting the results. This is partly because different PA behaviours may influence people’s perceptions of their specific surroundings differently [26]. This should be kept in mind for future studies. A further limitation might be the possibility that the neighbourhood environment within 10–15 min walking distance may already include environments of a different part of the city next to the residents’ own part of the city, especially in smaller city districts, so that a bias may have occurred. Also, although the city districts used are quite homogeneous within themselves, it is clear that variations exists and will detract from ideal conditions for this kind of study design. A strength is that we have controlled for age, sex, education and income in the multiple regression analyses. However, it is possible that also other variables, e.g., perceived physical and mental health, might affect environmental perceptions. Future studies should preferably aim at including such items. Moreover, we made use of a convenient sample in the present study where representativeness is not given and self-selection bias could occur. We therefore suggest that more studies using representative samples should look at the reported data using a similar approach. Finally, the present study uses a cross-sectional approach and thus prevents us from inferring causality.

## 5. Conclusions 

The present cross-sectional study lends support to the idea that residents with higher levels of transport and recreation PA may perceive their environment in a more “PA-favourable” way than residents with low PA living in the same neighbourhood. This finding adds to the research on the association between PA and the environment and strengthens the argument that we need more studies of this kind, as well as longitudinal studies looking at the association between PA and environmental perceptions in order to define causality. 
